# Cocaine Differentially Affects Mitochondrial Function Depending on Exposure Time

**DOI:** 10.3390/ijms26052131

**Published:** 2025-02-27

**Authors:** Sahar Wattad, Gabriella Bryant, Miriam Shmuel, Hannah L. Smith, Rami Yaka, Claire Thornton

**Affiliations:** 1 Institute for Drug Research (IDR), School of Pharmacy, Faculty of Medicine, The Hebrew University of Jerusalem, Jerusalem 91120, Israelmirishmuel55@gmail.com (M.S.); 2 Department of Comparative Biomedical Sciences, Royal Veterinary College, London NW1 0TU, UK

**Keywords:** cocaine, mitochondria, oxidative stress, bioenergetics

## Abstract

Cocaine use is a rising global concern, and increased use is accompanied by a significant increase in people entering treatment for the first time. However, there are still no complete therapies, and preclinical tools are necessary to both understand the action of cocaine and mitigate for its effects. Cocaine exposure rapidly impacts cellular and mitochondrial health, leading to oxidative stress. This study evaluated the effects of acute, repeated, and chronic cocaine exposure on C17.2 neural precursor cells. A single exposure to high concentrations of cocaine caused rapid cell death, with lower concentrations increasing markers of oxidative stress and mitochondrial dysfunction within 4 h of exposure. Alterations in cellular bioenergetics and mitochondrial fusion and fission gene expression (OPA1, DRP1) were also observed, which returned to baseline by 24 h after insult. Repeated exposure over 3 days reduced cell proliferation and spare mitochondrial respiratory capacity, suggesting compromised cellular resilience. Interestingly, chronic exposure over 4 weeks led to cellular adaptation and restoring mitochondrial bioenergetics and ATP production while mitigating for oxidative stress. These findings highlight the time-dependent cellular effects of cocaine, with initial toxicity and mitochondrial impairment transitioning to adaptive responses under chronic exposure.

## 1. Introduction

Cocaine is the second most common drug of abuse in the UK among adults aged 16–25 [1], with incidences in the UK being some of the highest in Western and Central Europe [2]. Cocaine causes both short- and long-term psychiatric and pathophysiological symptoms depending on dose and administration route, including increased heart rate, blood pressure, and body temperature, as well as agitation, convulsion/seizures, coma, and death [3]. The incidence of cocaine use is increasing in women; estrus enhances the effects of cocaine and, overall, the speed of transition to addiction as well as incidence of relapse is increased in women [4]. In this population, cocaine can, therefore, have indirect effects as the drug freely crosses the placenta (indeed placental metabolism may prolong fetal drug exposure) and prenatal cocaine exposure can induce deficits in cognitive function and behavior in offspring [5,6,7].

Currently, there are no complete therapies for cocaine addiction; the most effective therapies include psychosocial interventions such as cognitive behavioral therapy, but these are not universally effective [8]. Pharmacological studies have previously focused on either pharmacodynamic or pharmacokinetic approaches in order to facilitate therapy development. Pharmacokinetics research identified that peak serum concentrations of cocaine were difficult to assess, partly due to its rapid metabolism, but also because in real life settings, cocaine is often combined with other pharmacologically active substances [9]. As might be predicted, a user may potentially vary the dose themselves depending on the concentration of drug already on board [10], and the route of administration will also impact the exposure level [11]. Based on postmortem tissue as well as data from healthy volunteers in a clinical setting, serum concentrations can vary greatly, from recreational use (<10 µM) to binge use (<50 µM) to overdose (<100 µM and rarely 1000 µM [11,12,13]). Pharmacodynamics studies critically revealed that cocaine blocks the dopamine transporter (DAT), which is responsible for reabsorbing dopamine from the synaptic cleft back into the presynaptic neuron. Elevated dopamine levels result in prolonged activation and overstimulation of postsynaptic dopamine receptors, heightening euphoria [14]. Chronic cocaine use can lead to a sustained predominance of D1R signaling, contributing to compulsive drug-seeking behavior [15]. While D2 receptors are inhibitory, their activation is diminished relative to D1 receptors during cocaine use. This imbalance between D1 and D2 receptor signaling is thought to play a role in the transition from recreational use to addiction. Chronic use desensitizes these receptors, reducing their responsiveness over time and driving users to consume higher doses to achieve the same effect [15]. These observations initially led to the development of small molecules competing with the binding site of cocaine. However, as cocaine binds to the dopamine transporter [16], dopamine agonists competing for this site may have unwanted effects. In addition, cocaine has effects in the wider brain, and a multitarget treatment may be more effective. Computational modeling based on in vitro experimental data and human in vivo scanning identified an alternative approach in which cocaine was enzymatically degraded in the plasma, eliminating cocaine before it reached the brain [17]. Based on this model, cocaine hydrolase fused with human serum albumin was developed [18] and has already been tested in phase 2 clinical trials (although the outcome was very modest) [19]. Detailed molecular and cellular data are required to improve current theoretical models and to aid the development of new therapeutics.

The efficient function of mitochondria is important throughout the body but particularly in the brain, a site of intense energy usage, consuming 20% of available oxygen despite only accounting for 2% overall body weight [20]; in the developing brain, this energy demand increases further [21]. Terminally differentiated neurons have slow rates of mitochondrial turnover and, therefore, need to preserve healthy and functioning mitochondria throughout the entirety of their structures [22]. In addition, mitochondria in the brain have a longer half-life (24.4 days compared with liver [9.3 days] and heart [17.7 days], [23]), so mitochondrial quality control is critical. Mitochondrial health is dependent on a cycle of fusion and fission, with new mitochondria produced by biogenesis and impaired mitochondria degraded and recycled by mitophagy. Impairment of these balanced processes results in reduced cellular respiration/ATP production, increased reactive oxygen species (ROS) production, impaired calcium buffering, and ultimately cell death [24,25].

Cocaine exposure can disturb brain energy homeostasis through altered mitochondrial function and may target mitochondria directly. In vivo, repeated intraperitoneal (i.p.) injections of cocaine (20 mg/kg for 7 days) promoted mitochondrial fission through increased DRP1 activation in D1-medium spiny neurons of the nucleus accumbens (NAc) [26]. Changes in the expression of key regulators of mitochondrial dynamics (both fission and fusion) were also observed in the hippocampus and prefrontal cortex of rats following cocaine self-administration alongside increased ROS [27]. Drug seeking behavior is also blunted in cocaine-addicted rats after administration of the mitochondrial fission inhibitor-1 (Mdivi-1) [26]. Gene expression of markers of mitochondrial biogenesis were significantly increased in hippocampal mRNA from cocaine-treated rats (10–15 mg/kg, i.p.) that had undergone cocaine conditioning, extinction, and reinstatement of drug-seeking [28]. Transcriptomics analyses comparing acute, chronic, self-administration, and conditioned reward paradigms in the ventral tegmental area (VTA) of male mice identified that alterations in bioenergetic pathways were present in all conditions [29]. Supporting the concept of cocaine exposure regulating mitochondrial protein expression, activity of the transcription factor Egr3 was directed at nuclear-encoded genes of mitochondrial dynamics proteins in ex vivo rat samples from the NAc following cocaine exposure (20 mg/kg for 7 days) [30]. This study also provided evidence that human mitochondrial dynamics are altered in post mortem NAc samples of cocaine users [30].

In vitro, three-week exposure to high concentrations of cocaine (600 µM) resulted in increased mitochondrial fission accompanied by an increase in mitochondrial number and increased mitophagy in Neuro2A cells [31]. In primary mouse microglial cells, shorter exposures with lower concentrations of cocaine (1–100 µM, 24 h) also identified a dose-dependent decrease in mitochondrial membrane potential, along with increased expression of mitophagy markers [32]. Changes in the expression of mitochondrial biogenesis markers were also observed in cultured human astrocytes following a short exposure to low-dose cocaine (0.5 µM, 24 h) [33].

While in vitro studies have provided elegant data on the effects of cocaine on mitochondria, the majority use a single-dose paradigm, which may not be reflective of the actual cellular exposure. In this study, we aimed to investigate the impact of repeated, low-dose cocaine administration on cellular bioenergetics and ROS production in an immature neural cell line to identify differences between acute and chronic cocaine-mediated mitochondrial adaptations.

## 2. Results

### 2.1. A Single Exposure to Cocaine Rapidly Alters Cell Health and Mitochondrial Morphology

**Figure 1:**
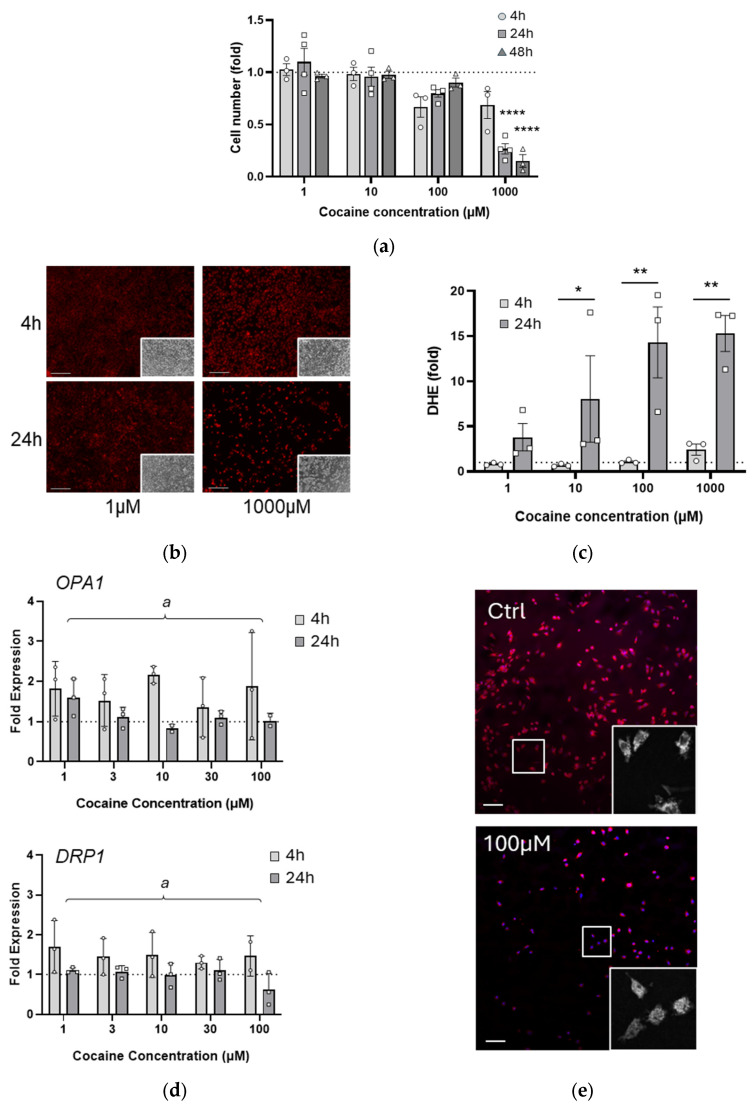
Single dose cocaine rapidly impairs cell health at high concentrations. (**a**) C17.2 cells were exposed to cocaine (1–1000 µM) and cell number assessed by Hoechst staining at 4, 24, and 48 h following treatment. **** *p* < 0.0001 compared with 4 h; (**b**,**c**) C17.2 cells were stained with DHE at 4 h and 24 h after single cocaine treatment (1–1000 µM, representative images shown in left panel). Thresholds were established on control images, and fluorescence above control expressed as a fold increase (right panel) Comparing 4 h and 24 h, * *p* = 0.0436, ** *p* = 0.0012 and ** *p* = 0.0015 for 10 µM 100 µM and 1000 µM respectively. Scale bar denotes 200 µm; (**d**) RNA was prepared from cells exposed to a single dose of cocaine at 4 h and 24 h following treatment. qRT-PCR was performed and expression of mitochondrial dynamics genes *OPA1* and *DRP1* determined. Data are shown normalized to *GAPDH* expression and to untreated C17.2 cells. Two-way ANOVA revealed an overall significant effect of time for both *OPA1* (^a^ *p* = 0.0198) and *DRP1* (^a^ *p* = 0.0029) expression; (**e**) C17.2 cells were treated with cocaine (100 µM, 4 h), stained with Hoechst and mitotracker red, and fixed and mounted on slides. Scale bar denotes 100 µm. Inset images show representative mitochondrial networks. For all graphs, data points (N) are representative of duplicate or triplicate measurements and are plotted as mean ± SEM. A dotted line represents values from untreated cells. Significance was determined using two-way ANOVA where overall significance was denoted by *a*; additional analyses were performed where appropriate using a Tukey post-hoc test.

To begin evaluating the cellular effects of repeated cocaine exposure, a single dose baseline was established. Our study used cocaine concentrations designed to mimic all stages of drug use from recreational to overdose (1–1000 µM [11,12]) and well within the range used in other in vitro studies (1 µM to 7 mM [31,34,35,36]). In addition, we chose to use the neural stem/precursor cell line C17.2 in order to evaluate whether exposure to cocaine in immature cells had a similar effect as that observed in more mature cells such as primary neurons [26]. C17.2 cells were treated with cocaine (1–1000 µM) and cell survival measured. No effects were observed at ≤10 µM, but there was an initial reduction in cell number following 100 µM treatment at 4 h which recovered by 24 h (Figure 1a). However, 1000 µM exposure resulted in rapid irreversible cell death (Figure 1a, *p* < 0.0001). Oxidative stress is also a key observation following in vitro cocaine exposure [37]. Cells were, therefore, subjected to cocaine and DHE fluorescence measured (Figure 1b). At 4 h, superoxide-related fluorescence was observed only in cells treated with 1000 µM; however, by 24 h post exposure, a significant increase in DHE was apparent for cells treated with 10 µM (*p* = 0.0436) and 100 µM (*p* = 0.0012) as well as 1000 µM (*p* = 0.0015) of cocaine (Figure 1c).

Dysfunctional mitochondria are a major source of pathological ROS; therefore, the effects of cocaine exposure on mitochondrial function were examined. As the previous experiment showed significant cell death with very high cocaine concentrations, the exposure range was now limited to a maximum of 100 µM, with additional intermediate concentrations reflecting approximately 3-fold increments. C17.2 cells were exposed to cocaine concentrations up to 100 µM, and genes involved in mitochondrial fusion (*OPA1*) and fission (*DRP1*) were assessed. There was an overall increase in gene expression for *OPA1* and *DRP1* by 4 h after exposure, which returned to baseline by 24 h (OPA1: *p* = 0.0198_time_, DRP1: *p* = 0.0029_time_, Figure 1d).

Following cocaine exposure (4 h, 100 µM), cells were fixed, and mitochondria were visualized with mitotracker red (Figure 1e). Alterations in mitochondrial morphology were observed and marked by a transition from interconnected mitochondria in control cells to a fragmented and punctate structure. These changes suggest disrupted mitochondrial dynamics, potentially indicative of increased fission.

### 2.2. Mitochondrial Function Is Transiently Impaired by Single Cocaine Exposure

**Figure 2:**
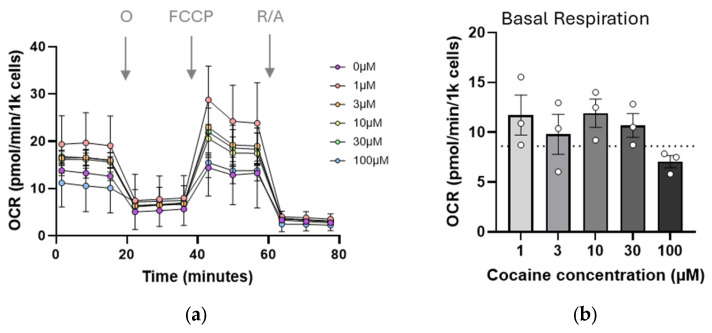

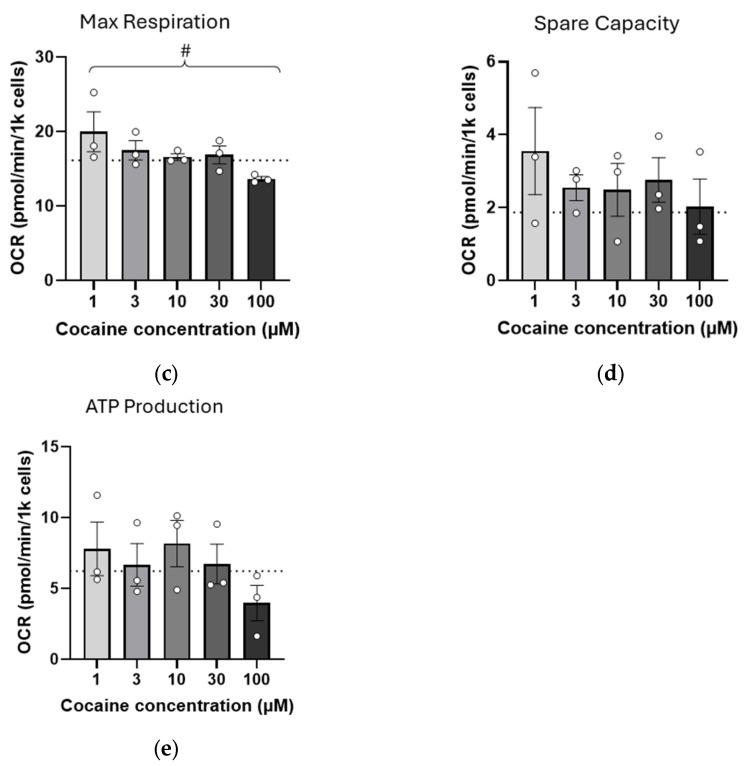
Single dose cocaine transiently impairs mitochondrial bioenergetics. C17.2 cells were treated with cocaine (1–100 µM), and a bioenergetic profile was established using a Seahorse mito stress test (**a**). From the profile, mitochondrial basal respiration (**b**), maximal respiration (^#^ *p* = 0.0162 for trend) (**c**), spare respiratory capacity (**d**), and ATP production (**e**) were calculated. For all graphs, data points (N) are representative of 5–8 measurements and are plotted as mean ± SEM. A dotted line represents values from untreated cells. Significance was determined using one-way ANOVA followed by analysis for trend. O: Oligomycin, FCCP: Carbonyl cyanide-p-trifluoromethoxyphenylhydrazone, R/A: Rotenone/Antimycin A.

As mitochondrial morphology appeared altered by cocaine exposure, mitochondrial bioenergetics were assessed using the Seahorse mito stress assay. Cells were exposed to cocaine, and 4 h later, were treated with oligomycin, FCCP, and a combination of rotenone and antimycin A to target ATP synthase, proton gradient, and complex I function, respectively (Figure 2). Although the magnitude of the observed changes was small, a universal negative trend was apparent in all conditions tested (Figure 2b–e). This trend reached significance for maximal respiration (*p* = 0.0162, Figure 2c), where increasing concentrations of cocaine progressively decreased respiration. Mitochondrial bioenergetics were also measured at 24 h post exposure. However, no effects of the drug were observed for any of the above metrics even at higher concentrations, suggesting that the impact of single dose cocaine on mitochondrial function was transient.

### 2.3. Repeated (Three-Day) Exposure to Cocaine Reduces Mitochondrial Reserve

**Figure 3:**
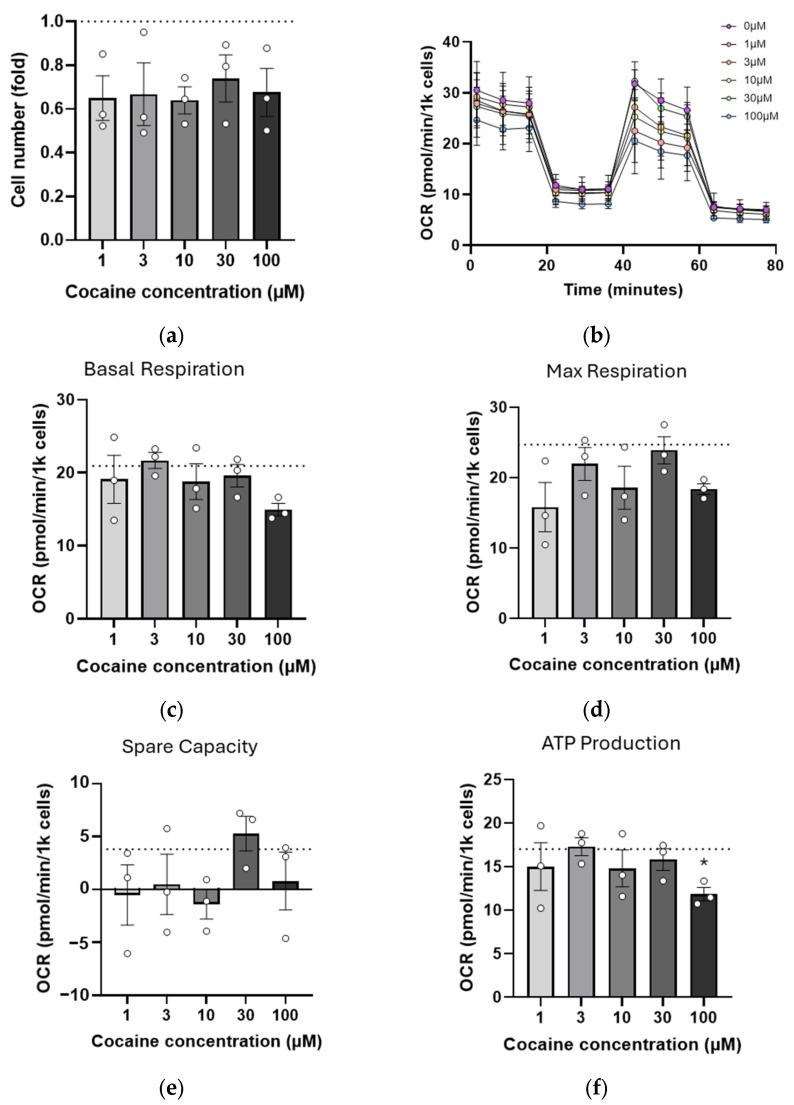
Three-day repeated low dose cocaine exhausts spare respiratory capacity. C17.2 cells were exposed to daily cocaine doses (1–100 µM) for three days and analyzed 24 h following the final treatment. (**a**) Cells were stained with Hoechst and counted using Fiji v1.54, as previously described. (**b**–**f**) Treated cells were analyzed using a mito stress test for their bioenergetic profile (**b**). Values for mitochondrial basal respiration (**c**), maximal respiration (**d**), spare respiratory capacity (**e**), and ATP production ((**f**), * *p* = 0.0106) were established. For all graphs, data points (N) comprise means of 4–6 measurements ± SEM. A dotted line represents values from untreated cells. A significant difference was determined using one-way ANOVA followed by Tukey post-hoc analysis where appropriate.

As it is unlikely that drug use occurs in isolation, a repeated cocaine dosing paradigm was tested, treating cells daily for 3 days before analysis on day 4. Following 3 days of cocaine exposure (1–100 µM), cell numbers were noticeably reduced compared with the control, regardless of cocaine concentration (Figure 3a). Three-day cocaine exposure did not result in changes in mitochondrial morphology or mitochondrial fission/fusion gene expression (). Mitochondrial bioenergetics were again assessed using Seahorse mito stress test (Figure 3b). Basal respiration remained largely unchanged (Figure 3c), but, although maximal respiration was below control levels, the negative trend seen following single cocaine exposure was now absent (Figure 3d). Interestingly, spare respiratory capacity was also largely absent in these cells at all concentrations tested (Figure 3e). ATP production remained impaired at the highest dose compared with untreated cells (*p* = 0.0106, Figure 3f).

### 2.4. Chronic (4-Week) Exposure to Cocaine Results in Adaptation, Recovering Cell Health

**Figure 4:**
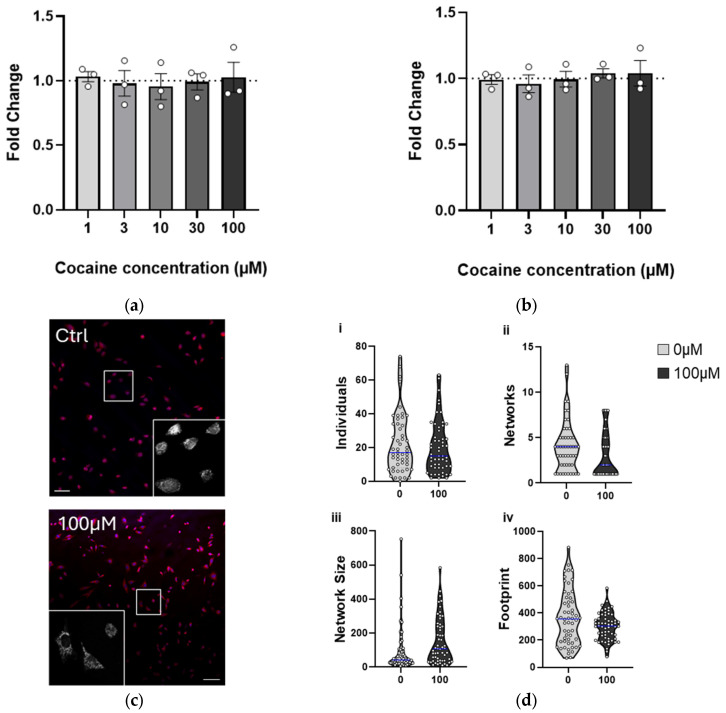
Four-week repeated exposure to cocaine changes mitochondria morphology. C17.2 cells were treated with cocaine 2–3 times per week for 4 weeks; cell number (**a**) and ROS production (**b**) were analyzed, as previously described. Data points (N) are representative of duplicate or triplicate measurements and are plotted as mean ± SEM. A dotted line represents values from untreated cells. (**c**) C17.2 cells were treated with cocaine over four weeks, stained with mitotracker red, and fixed and mounted in medium containing DAPI. Scale bar denotes 100 µm. Inset images show representative mitochondrial networks. (**d**) Images from c were analyzed using a Mitochondrial Network Analysis (MiNA) plugin in Fiji. Parameters measured included number of individual mitochondria (**i**), mitochondrial network number (**ii**), network size (**iii**), and mitochondrial footprint (**iv**). Data points represent individual cells from three independent experiments and have a non-parametric distribution. Data are shown in violin plots for distribution, with the median highlighted (blue).

Finally, to reflect a chronic exposure paradigm, the study duration was extended. Cells were treated with cocaine two to three times per week for 4 weeks and analyzed 24 h after the final treatment. In contrast with the previous treatments, prolonged exposure was not detrimental to cell survival or proliferation (Figure 4a), and ROS production remained unaffected (Figure 4b), suggesting that the cells had adapted to the presence of cocaine.

Morphological analysis of cocaine-treated cells (100 µM) revealed no gross changes, and mitochondrial number remained the same (Figure 4c,d(i)). More detailed mitochondrial network analyses suggested that prolonged cocaine exposure resulted in a decrease in the number of networks, but those networks were increasingly complex (Figure 4d(ii,iii)). Mitochondria also appeared more clustered around the nucleus following cocaine treatment (Figure 4c), and this was reflected in the coverage within the cell (Figure 4d(iv)).

**Figure 5:**
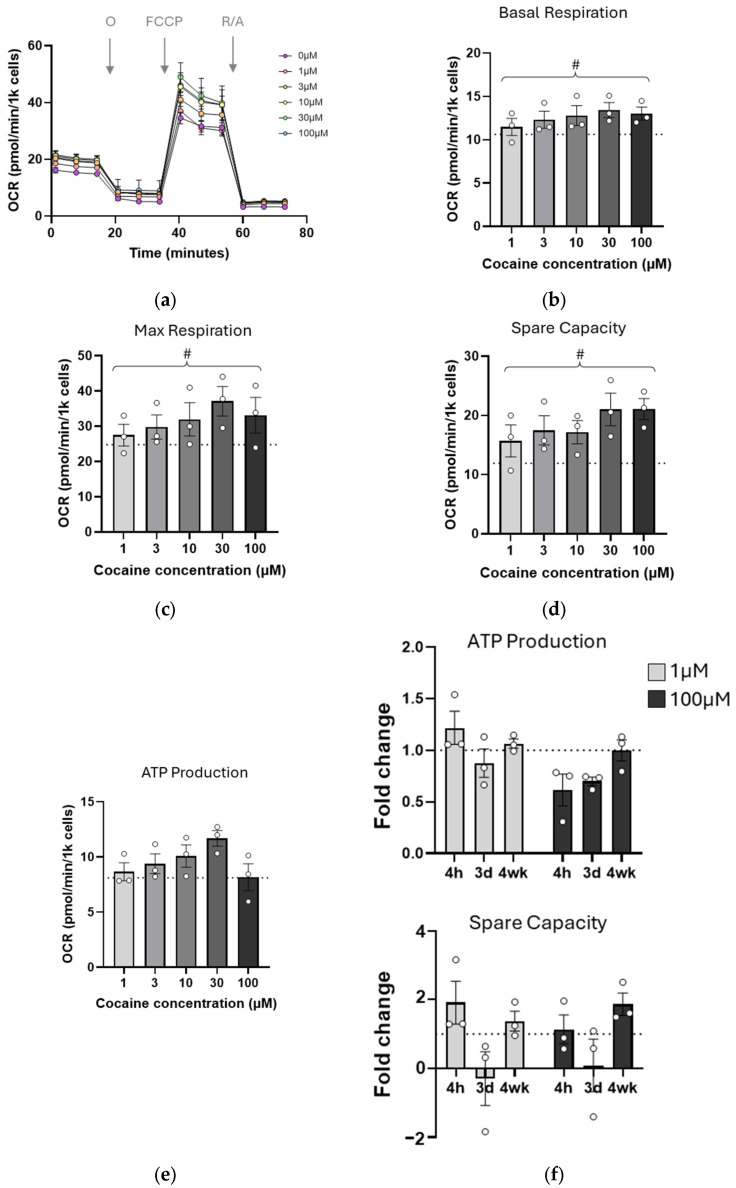
Four-week cocaine exposure increases mitochondrial output. (**a**) C17.2 cells treated with cocaine for four weeks were analyzed using a mito stress test for their bioenergetic profile. Values for mitochondrial basal respiration ((**b**) ^#^ *p* = 0.0313), maximal respiration ((**c**) ^#^ *p* = 0.0463), spare respiratory capacity ((**d**) ^#^ *p* = 0.0086), and ATP production (**e**) were established. (**f**) A summary comparison of the ATP production and spare respiratory capacity over the time period of the study. For all graphs, data points (N) comprise means of 4 measurements ± SEM. A dotted line represents values from untreated cells. A significant difference was determined using one-way ANOVA. O: Oligomycin, FCCP: Carbonyl cyanide-p-trifluoromethoxyphenylhydrazone, R/A: Rotenone/Antimycin A.

Mitochondrial bioenergetics were also measured (Figure 5a). Surprisingly, the negative trends which were apparent at single and 3-day treatments were no longer observed, and all metrics tested showed ranges above control (Figure 5b–e). There were upward and significant trends in basal respiration (Figure 5b, *p* = 0.0313) and maximal respiration (Figure 5c, *p* = 0.0463) as well as restoration of spare respiratory capacity (Figure 5d,f, *p* = 0.0086). ATP levels were also increased above control values (1–30 µM) but limited at the highest concentration (Figure 5e,f, 100 µM).

## 3. Discussion

In this study, we aimed to identify changes in cell health and bioenergetics in an immature cell line in response to cocaine exposure, comparing acute (single dose) and chronic (4-week dosing) effects. We found that, while acute exposure impacts cell viability, ROS, and mitochondrial function negatively, chronic effects become more subtle, suggesting that the cells may be adapting to the cocaine-rich environment.

The concentration of cocaine used in in vitro studies to reflect the in vivo condition is always approximated. We examined other in vitro studies to determine our dose range and identified that there was wide variation in the doses used, ranging from 1 µM to 7 mM, dependent on cell type [31,34,35,36]. In vivo, in humans, and in preclinical models, cocaine is rapidly metabolized, and therefore it is difficult to gain a measure of peak serum concentrations, and there can be large intersubject variation [11]. Measurements in post-mortem samples of habitual users have provided a range of serum concentrations from 0.03 µM to 70 µM, and there are reports of human overdose cases where cocaine concentrations were determined around 1000 µM [38,39,40]. Computational modeling of plasma and brain concentrations of cocaine with PET imaging of ^11C^cocaine showed that brain levels correlate with plasma levels, and the half-life is directly proportional to the initial concentration in plasma [17]. Here, we initially used a range from 1–1000 µM, but due to the increased cell death at the highest concentration, we performed the majority of our experiments with an upper limit of 100 µM, reflective of the published serum concentrations. In addition, we chose to use an immature neural precursor/stem cell line C17.2. Previous in vitro studies have utilized primary neurons, glia, or neuronal cell lines [34], but information on the effects of cocaine on mitochondria in primary neural stem cells or the developing brain is still lacking, although there are promising studies in developing zebrafish models where cocaine exposure at the larval stage results in disrupted brain development, gene expression, and impaired dopaminergic signaling due to downregulation of expression of the dopamine receptor [41,42,43]. These data support the choice of cocaine concentration and cell type used for this study.

Our experiments showed that cell viability was impaired rapidly after initial cocaine exposure but that this occurred only in concentrations of 100 µM and above. This rapid death is likely due to necrosis, as apoptotic or other programmed cell death would usually occur later after insult. No cell loss was observed after long-term exposure, in line with previously published observations examining 3-week exposure of Neuro2a cells to 600 µM cocaine [31]. The in vitro response to cocaine is also likely to depend on cell type, as Neuro2a cells are resistant to cocaine toxicity even at 1 mM concentrations [31,44]. This resistance may be due to the cell being able to mount a robust antioxidant defense (including *Nrf2*-mediated increases in catalase, glutathione, and glutathione peroxidase [44]), which may partly be the case for C17.2 cells. We recently compared *Nrf2* expression in C17.2 cells and BV2 cells and found increased expression in the former, providing C17.2 cells with the means to overcome the environmental insult [45]. This may explain why the increases in cocaine-mediated ROS observed after single dose administration are not apparent in repeated dosing paradigms, where there has been time to increase the antioxidant response. However, this antioxidant upregulation may not be permanent, as impaired antioxidant capacity is reported in cocaine-dependent adults; blood samples revealed a significant reduction in superoxide dismutase contributing to a halving of total antioxidant capacity [46]. In vitro [47,48] and in preclinical cocaine-exposed models [47,49], antioxidant treatment has resulted in a reduction in oxidative stress and improved outcomes in behavioral tests such as cocaine-mediated psychomotor sensitization and cocaine-conditioned place preference.

Impairments of mitochondrial function are reported in a number of cell types following single cocaine exposure [34]. Here, we found that ATP production is noticeably reduced in cells exposed to 100 µM cocaine compared with all other concentrations, although the levels start to recover to control levels in the 4-week paradigm. Similarly, there is a significant negative trend in maximal respiration following single dose exposure that is reversed after 4 weeks of cocaine treatment. Spare respiratory capacity is also impacted; single dose cocaine reduces and 3 days of treatment almost eliminates spare capacity, but this is reversed in the 4-week data. The lack of mitochondrial reserve in the 3-day paradigm may indicate that the cell has been drawing on all its resources to maintain essential biological functions following a prolonged period of stress [50]. As oxidative stress is not observed following 4-week cocaine exposure, it is likely that this heavy demand for energy has been incorporated through an adaptive response, potentially through altered gene expression. Comprehensive studies have reported differential gene expression in response to acute and chronic cocaine exposure in both rodent and human brain, highlighting neuroinflammation and mitochondrial pathway adaptation, among others [29,51,52]. In line with these findings, cocaine-mediated epigenetic modifications have been shown to directly and indirectly influence mitochondria gene expression and function [53]. Cocaine exposure in human astrocytes and rodent models altered the expression of various epigenetic factors, including decreasing the expression of DNA methyltransferases (DNMT)1 and DMNT3a, as well as ten-eleven translocation enzymes (TET)1–3 [54]. Mitochondrially localized DMNT expression is changed after cocaine exposure, and these cocaine-induced epigenetic modifications of the mitochondrial genome result in impaired mitochondrial gene transcription and alterations in oxidative phosphorylation [54,55]. Again, it is critical that cell type, length of exposure, and brain region need to be considered, as cocaine-mediated epigenetic modification was increased in the nucleus accumbens [56], but some of the subsequent epigenetic effects of cocaine exposure in this brain region were observed in D2 receptor but not D1 receptor-expressing neurons [53,57]. Cocaine can also influence the methylation of nuclear genes involved in mitochondrial biogenesis (*PGC1α*, *NRF2* [30]), suggesting an influence on cellular mitochondrial load.

We observed a trend towards increased mitochondrial network complexity following prolonged cocaine exposure, in contrast to the fission-like morphology observed following acute treatments with cocaine. Such reticulated networks would facilitate increased ETC function and promote ATP production. However, an intriguing candidate for mediating an alternative adaptive response is proline oxidase (POX), an enzyme localized at the inner mitochondrial membrane responsible for the catabolism of proline to pyrroline-5-carboxylate [58]. POX activity generates electrons, fueling ROS or ATP production and has been previously shown to increase ATP production in SH-SY5Y cells in response to cocaine exposure [59]. As enhanced ROS is not evident at the 4-week timepoint, it is possible that this stress-responsive pathway has been triggered, favoring ATP production. Long-term cocaine self-administration in rats has also identified neuroadaptations; on abstinence, decreased metabolic activity is observed in the NAc, prefrontal cortex, and anterior cingulate cortex [60,61,62]. Longitudinal studies during abstinence also identified increased metabolic activity in the hippocampus [62]. Although our work is in vitro, it supports the idea that where brain cells have adapted positively to the presence of cocaine, its removal from the environment triggers a more significant response in that region. It would be interesting to determine the metabolic profile of cells in the 4-week paradigm after a period without cocaine exposure.

## 4. Limitations of the Study

As with any research, our study has limitations. ROS detection was based on DHE fluorescence, which primarily measures overall superoxide levels. Using—an assay to specifically detect mitochondrial ROS would provide a more precise understanding of oxidative stress sources. Moreover, mitochondrial morphology analysis relied on fixed-cell imaging and MiNA network analysis, which limits the ability to capture dynamic mitochondrial changes. Live-cell imaging with time-lapse microscopy could provide further insights into real-time alterations in fusion, fission, and trafficking. Although our study is one of the few to address the impact of cocaine on mitochondria in immature cells, our study is limited in its findings through the use of a neural stem/precursor cell line, C17.2. A number of studies have utilized primary neurons or neuronal cell lines, but to our knowledge, information on the effects of cocaine on mitochondria in primary neural stem cells or the developing brain is still lacking. Additionally, although we investigated mitochondrial respiration across time, we have not defined the mechanism leading to the bioenergetics changes observed. Our previous use of multiomics approaches [63] has provided a comprehensive snapshot of mitochondrial dysfunction following acute cocaine administration in 6-week old mice (roughly equivalent to human age 12 years). Taking a similar multiomics approach to determining pathways altered in the developing fetal brain in offspring of a cocaine-addicted dam would add significantly to the in vitro work described here.

## 5. Materials and Methods

### 5.1. Cell Culture and Cocaine Treatment

Mouse C17.2 cerebellar neural precursor cell line (ECACC 07062902, male-derived, passages 12–25) and all media/supplements were from Thermo Fisher Scientific (Loughborough, UK). Cells were grown at 37 °C, 5% CO_2_ in Dulbeccos’s modified eagle medium (DMEM) supplemented with 10% heat inactivated fetal bovine serum (FBS), 1% penicillin/streptomycin and passaged every 3–4 days. Cocaine hydrochloride (10 mM, Sigma, St. Louis, MO, USA) was freshly prepared in sterile PBS, filter sterilized, and diluted in DMEM to the appropriate final concentration (1–100 µM). Chronic exposure was achieved by growing cells in medium containing cocaine for 4 weeks; the cocaine-containing medium was refreshed 2–3 times per week.

### 5.2. ROS Analysis

C17.2 cells were treated with DMEM containing dihydroethidium (DHE, Sigma, 10 µM) and incubated for 30 min at 37 °C, 5% CO_2_. Cells were washed with DMEM to remove unincorporated dye and imaged using a Biotek Cytation platereader (Agilent, Santa clara, CA, USA). Images were imported into Fiji (Image Jv 1.54), and control images of untreated cells were used to establish threshold values. Once all images were thresholded, the number of cells with fluorescence above control were counted.

### 5.3. Nuclear and Mitochondrial Imaging

Hoechst 33,342 (10 µg/mL final concentration, Sigma, St. Louis, MO, USA) and Mitotracker Red CMXROS (70 nM, Thermo Fisher) were added to cells seeded on coverslips. Following incubation (37 °C, 5% CO_2_, 30 min protected from light), cells were washed and fixed with 4% paraformaldehyde (PFA, 10 min). Coverslips were washed then mounted on slides using Prolong™ Diamond Antifade Mountant (Thermo Fisher). Stained cells were imaged using Nikon confocal AXR microscope (Nikon Instruments Inc., Melville, NY, USA) using 60× objective.

### 5.4. Quantitative Real-Time PCR

RNA was extracted from treated and control C17.2 cells using RNeasy isolation kit (QIAGEN, Hilden, Germany) according to the manufacturer’s instructions. Purified RNA was eluted in RNase free water before integrity and purity were verified by ND-1000 spectrophotometer (NanoDrop Technologies Inc., Thermo Fisher Scientific). cDNA was synthesized from 1 µg total RNA using qScript^®^ cDNA Synthesis Kit (Quantabio, Beverly, MA, USA) and the following parameters: (i) 22 °C, 5 min (1 cycle) (ii) 42 °C, 30 min (1 cycle) (iii) 85 °C, 5 min (1 cycle) (iv) 4 °C (hold). Polymerase chain reaction (PCR) assay was carried out using the following TaqMan gene expression assays: Drp1 (Mm01342903), GAPDH (Mm99999915), and OPA1 (Mm01349707_g1) in StepOnePlus real-time PCR system (Applied Biosystems, Foster City, CA, USA) on a fast mode (40 amplification cycles of 95 °C, 15 s, 60 °C, 1 min). Relative gene expression was analyzed using the 2^ΔΔCt^ method [64], and the data were normalized both to GAPDH and to control, untreated samples.

### 5.5. Oxygen Consumption Rate (OCR)

Cells were seeded on 96-well Seahorse plates (Agilent) at a concentration of 10,000–20,000 cells per well depending on whether the assay was performed at 48 h or 24 h post plating, respectively. XF DMEM assay medium (Agilent) was prepared containing 10 mM glucose, 1 mM pyruvate and 2 mM glutamine and used to dilute the mito stress test (Agilent) reagents for final concentrations of 1.5 µM oligomycin, 1 µM FCCP and 0.5 µM rotenone/0.5 µM antimycin A. The assay was performed on a Seahorse XF Pro analyzer (Agilent). For normalizing the assay data, the cell number per well was calculated by incubation with Hoechst (0.2 µg/mL, 30 min, 37 °C). Following incubation, wells were washed, and fluorescent nuclei were counted using a SparkCyto plate reader (Tecan, Reading, UK). All data were input and analyzed using the Seahorse Analytics software (https://seahorseanalytics.agilent.com/#; accessed on 1 September 2024).

### 5.6. Statistics

Statistical analysis was conducted using Prism Software (v10, GraphPad, Boston, MA, USA). Data were evaluated for normality (Shapiro–Wilk) and assessed using one-way or two-way ANOVA and appropriate post hoc tests (shown in figure legends). A *p*-value of <0.05 was deemed significant. For C17.2 analysis, biological replicates were defined as experiments on different passages of cells, and each biological replicate comprised a minimum of two technical replicates.

## 6. Conclusions

In this study, we show that cocaine has different effects on mitochondrial function in neural precursor stem cell line C17.2, dependent on the length of exposure. Longer exposures to low dose cocaine (≤30 µM) results in increased ATP production in the absence of superoxide production (in contrast with acute exposure), although this falls away at 100 µM exposure. Prolonged exposure may, therefore, result in adaptive responses; such prolonged exposure may mimic that experienced in utero. Future multiomics analyses will provide a clearer picture of the bioenergetic adaptations that occur in the developing brain in response to maternal cocaine use and may be critical in any strategies designed to promote drug abstinence.

## Data Availability

The data presented in this study are available on request from the corresponding authors.

## Abbreviations

ANOVA, analysis of variance; ATP, adenosine triphosphate; Ctrl, control; DHE, dihydroethidium; DMEM, Dulbecco’s modified eagle medium; DRP1, dynamin-related protein 1; ETC, electron transport chain; FCCP, carbonyl cyanide-p-trifluoromethoxyphenylhydrazone; GAPDH, glyceraldehyde-3-phosphate dehydrogenase; NAc, nucleus accumbens; O, oligomycin; OCR, oxygen consumption rate; OPA1, optic atrophy 1; PET, positron emission tomography; POX, proline oxidase; R/A, rotenone/antimycin A; ROS, reactive oxygen species; SEM, standard error of the mean; VTA, ventral tegmental area.

## References

[1] UKGov United Kingdom Drug Situation 2019: Focal Point Annual Report. https://www.gov.uk/government/publications/united-kingdom-drug-situation-focal-point-annual-report.

[2] UNODC (2022). World Drug Report 2022.

[3] Morton W.A. (1999). Cocaine and Psychiatric Symptoms. Prim. Care Companion J. Clin. Psychiatry.

[4] Calipari E.S., Juarez B., Morel C., Walker D.M., Cahill M.E., Ribeiro E., Roman-Ortiz C., Ramakrishnan C., Deisseroth K., Han M.-H. (2017). Dopaminergic dynamics underlying sex-specific cocaine reward. Nat. Commun..

[5] De Giovanni N., Marchetti D. (2012). Cocaine and its metabolites in the placenta: A systematic review of the literature. Reprod. Toxicol..

[6] Richardson G.A., De Genna N.M., Willford J.A., Goldschmidt L. (2024). Pathways from prenatal cocaine exposure to adult substance use and behavior. Neurotoxicol. Teratol..

[7] Grewen K., Burchinal M., Vachet C., Gouttard S., Gilmore J.H., Lin W., Johns J., Elam M., Gerig G. (2014). Prenatal cocaine effects on brain structure in early infancy. NeuroImage.

[8] Kampman K.M. (2019). The treatment of cocaine use disorder. Sci. Adv..

[9] Solimini R., Rotolo M.C., Pellegrini M., Minutillo A., Pacifici R., Busardo F.P., Zaami S. (2017). Adulteration Practices of Psychoactive Illicit Drugs: An Updated Review. Curr. Pharm. Biotechnol..

[10] Zimmer B.A., Dobrin C.V., Roberts D.C. (2011). Brain-cocaine concentrations determine the dose self-administered by rats on a novel behaviorally dependent dosing schedule. Neuropsychopharmacol. Off. Publ. Am. Coll. Neuropsychopharmacol..

[11] Heard K., Palmer R., Zahniser N.R. (2008). Mechanisms of acute cocaine toxicity. Open Pharmacol. J..

[12] Barnett G., Hawks R., Resnick R. (1981). Cocaine pharmacokinetics in humans. J. Ethnopharmacol..

[13] Thornton C., Grad E., Yaka R. (2021). The role of mitochondria in cocaine addiction. Biochem. J..

[14] Verma V. (2015). Classic Studies on the Interaction of Cocaine and the Dopamine Transporter. Clin. Psychopharmacol. Neurosci..

[15] Park K., Volkow N.D., Pan Y., Du C. (2013). Chronic cocaine dampens dopamine signaling during cocaine intoxication and unbalances D1 over D2 receptor signaling. J. Neurosci..

[16] Beuming T., Kniazeff J., Bergmann M.L., Shi L., Gracia L., Raniszewska K., Newman A.H., Javitch J.A., Weinstein H., Gether U. (2008). The binding sites for cocaine and dopamine in the dopamine transporter overlap. Nat. Neurosci..

[17] Zheng F., Zhan C.G. (2012). Modeling of pharmacokinetics of cocaine in human reveals the feasibility for development of enzyme therapies for drugs of abuse. PLoS Comput. Biol..

[18] Zheng F., Jin Z., Deng J., Chen X., Zheng X., Wang G., Kim K., Shang L., Zhou Z., Zhan C.-G. (2022). Development of a Highly Efficient Long-Acting Cocaine Hydrolase Entity to Accelerate Cocaine Metabolism. Bioconjugate Chem..

[19] Gilgun-Sherki Y., Eliaz R.E., McCann D.J., Loupe P.S., Eyal E., Blatt K., Cohen-Barak O., Hallak H., Chiang N., Gyaw S. (2016). Placebo-controlled evaluation of a bioengineered, cocaine-metabolizing fusion protein, TV-1380 (AlbuBChE), in the treatment of cocaine dependence. Drug Alcohol. Depend..

[20] Raichle M.E., Gusnard D.A. (2002). Appraising the brain’s energy budget. Proc. Natl. Acad. Sci. USA.

[21] Kuzawa C.W., Chugani H.T., Grossman L.I., Lipovich L., Muzik O., Hof P.R., Wildman D.E., Sherwood C.C., Leonard W.R., Lange N. (2014). Metabolic costs and evolutionary implications of human brain development. Proc. Natl. Acad. Sci. USA.

[22] Misgeld T., Schwarz T.L. (2017). Mitostasis in Neurons: Maintaining Mitochondria in an Extended Cellular Architecture. Neuron.

[23] Menzies R.A., Gold P.H. (1971). The Turnover of Mitochondria in a Variety of Tissues of Young Adult and Aged Rats. J. Biol. Chem..

[24] Sprenger H.G., Langer T. (2019). The Good and the Bad of Mitochondrial Breakups. Trends Cell Biol..

[25] Yapa N.M.B., Lisnyak V., Reljic B., Ryan M.T. (2021). Mitochondrial dynamics in health and disease. FEBS Lett..

[26] Chandra R., Engeln M., Schiefer C., Patton M.H., Martin J.A., Werner C.T., Riggs L.M., Francis T.C., McGlincy M., Evans B. (2017). Drp1 Mitochondrial Fission in D1 Neurons Mediates Behavioral and Cellular Plasticity during Early Cocaine Abstinence. Neuron.

[27] Sadakierska-Chudy A., Kotarska A., Frankowska M., Jastrzebska J., Wydra K., Miszkiel J., Przegalinski E., Filip M. (2017). The Alterations in Mitochondrial DNA Copy Number and Nuclear-Encoded Mitochondrial Genes in Rat Brain Structures after Cocaine Self-Administration. Mol. Neurobiol..

[28] Pati S., Angel P., Drake R.R., Wagner J.J., Cummings B.S. (2019). Lipidomic changes in the rat hippocampus following cocaine conditioning, extinction, and reinstatement of drug-seeking. Brain Behav..

[29] Campbell R.R., Chen S., Beardwood J.H., López A.J., Pham L.V., Keiser A.M., Childs J.E., Matheos D.P., Swarup V., Baldi P. (2021). Cocaine induces paradigm-specific changes to the transcriptome within the ventral tegmental area. Neuropsychopharmacol. Off. Publ. Am. Coll. Neuropsychopharmacol..

[30] Cole S.L., Chandra R., Harris M., Patel I., Wang T., Kim H., Jensen L., Russo S.J., Turecki G., Gancarz-Kausch A.M. (2021). Cocaine-induced neuron subtype mitochondrial dynamics through Egr3 transcriptional regulation. Mol. Brain.

[31] Funakoshi T., Furukawa M., Aki T., Uemura K. (2019). Repeated exposure of cocaine alters mitochondrial dynamics in mouse neuroblastoma Neuro2a. Neurotoxicology.

[32] Thangaraj A., Periyasamy P., Guo M.L., Chivero E.T., Callen S., Buch S. (2020). Mitigation of cocaine-mediated mitochondrial damage, defective mitophagy and microglial activation by superoxide dismutase mimetics. Autophagy.

[33] Sivalingam K., Cirino T.J., McLaughlin J.P., Samikkannu T. (2021). HIV-Tat and Cocaine Impact Brain Energy Metabolism: Redox Modification and Mitochondrial Biogenesis Influence NRF Transcription-Mediated Neurodegeneration. Mol. Neurobiol..

[34] Pereira S.P., Cunha-Oliveira T., Preedy V.R. (2017). Role of Mitochondria on the Neurological Effects of Cocaine. The Neuroscience of Cocaine.

[35] Cunha-Oliveira T., Rego A.C., Morgadinho M.T., Macedo T., Oliveira C.R. (2006). Differential cytotoxic responses of PC12 cells chronically exposed to psychostimulants or to hydrogen peroxide. Toxicology.

[36] Badisa R.B., Goodman C.B. (2012). Effects of chronic cocaine in rat C6 astroglial cells. Int. J. Mol. Med..

[37] Beiser T., Yaka R. (2019). The Role of Oxidative Stress in Cocaine Addiction. J. Neurol. Neuromedicine.

[38] Karch S.B., Stephens B., Ho C.H. (1998). Relating cocaine blood concentrations to toxicity--an autopsy study of 99 cases. J. Forensic Sci..

[39] Mittleman R.E., Wetli C.V. (1984). Death caused by recreational cocaine use. An update. JAMA.

[40] Peretti F.J., Isenschmid D.S., Levine B., Caplan Y.H., Smialek J.E. (1990). Cocaine fatality: An unexplained blood concentration in a fatal overdose. Forensic Sci. Int..

[41] Shang E.H., Zhdanova I.V. (2007). The circadian system is a target and modulator of prenatal cocaine effects. PLoS ONE.

[42] López Patiño M.A., Yu L., Yamamoto B.K., Zhdanova I.V. (2008). Gender differences in zebrafish responses to cocaine withdrawal. Physiol. Behav..

[43] Riley E., Maymi V., Pawlyszyn S., Yu L., Zhdanova I.V. (2018). Prenatal cocaine exposure disrupts the dopaminergic system and its postnatal responses to cocaine. Genes. Brain Behav..

[44] Badisa R.B., Wi S., Jones Z., Mazzio E., Zhou Y., Rosenberg J.T., Latinwo L.M., Grant S.C., Goodman C.B. (2018). Cellular and molecular responses to acute cocaine treatment in neuronal-like N2a cells: Potential mechanism for its resistance in cell death. Cell Death Discov..

[45] Curel C.J.M., Nobeli I., Thornton C. (2024). Leflunomide Treatment Does Not Protect Neural Cells following Oxygen-Glucose Deprivation (OGD) In Vitro. Cells.

[46] Walker J., Winhusen T., Storkson J.M., Lewis D., Pariza M.W., Somoza E., Somoza V. (2014). Total antioxidant capacity is significantly lower in cocaine-dependent and methamphetamine-dependent patients relative to normal controls: Results from a preliminary study. Hum. Psychopharmacol..

[47] Numa R., Kohen R., Poltyrev T., Yaka R. (2008). Tempol diminishes cocaine-induced oxidative damage and attenuates the development and expression of behavioral sensitization. Neuroscience.

[48] Numa R., Baron M., Kohen R., Yaka R. (2011). Tempol attenuates cocaine-induced death of PC12 cells through decreased oxidative damage. Eur. J. Pharmacol..

[49] Beiser T., Numa R., Kohen R., Yaka R. (2017). Chronic treatment with Tempol during acquisition or withdrawal from CPP abolishes the expression of cocaine reward and diminishes oxidative damage. Sci. Rep..

[50] Marchetti P., Fovez Q., Germain N., Khamari R., Kluza J. (2020). Mitochondrial spare respiratory capacity: Mechanisms, regulation, and significance in non-transformed and cancer cells. FASEB J..

[51] Zhou Z., Yuan Q., Mash D.C., Goldman D. (2011). Substance-specific and shared transcription and epigenetic changes in the human hippocampus chronically exposed to cocaine and alcohol. Proc. Natl. Acad. Sci. USA.

[52] Mews P., Cunningham A.M., Scarpa J., Ramakrishnan A., Hicks E.M., Bolnick S., Garamszegi S., Shen L., Mash D.C., Nestler E.J. (2023). Convergent abnormalities in striatal gene networks in human cocaine use disorder and mouse cocaine administration models. Sci. Adv..

[53] Vaillancourt K., Ernst C., Mash D., Turecki G. (2017). DNA Methylation Dynamics and Cocaine in the Brain: Progress and Prospects. Genes.

[54] Doke M., Jeganathan V., McLaughlin J.P., Samikkannu T. (2020). HIV-1 Tat and cocaine impact mitochondrial epigenetics: Effects on DNA methylation. Epigenetics.

[55] Samikkannu T., Atluri V.S., Nair M.P. (2016). HIV and Cocaine Impact Glial Metabolism: Energy Sensor AMP-activated protein kinase Role in Mitochondrial Biogenesis and Epigenetic Remodeling. Sci. Rep..

[56] Anier K., Malinovskaja K., Aonurm-Helm A., Zharkovsky A., Kalda A. (2010). DNA Methylation Regulates Cocaine-Induced Behavioral Sensitization in Mice. Neuropsychopharmacol. Off. Publ. Am. Coll. Neuropsychopharmacol..

[57] Engmann O., Labonté B., Mitchell A., Bashtrykov P., Calipari E.S., Rosenbluh C., Loh Y.-H.E., Walker D.M., Burek D., Hamilton P.J. (2017). Cocaine-Induced Chromatin Modifications Associate with Increased Expression and Three-Dimensional Looping of Auts2. Biol. Psychiatry.

[58] Chalecka M., Kazberuk A., Palka J., Surazynski A. (2021). P5C as an Interface of Proline Interconvertible Amino Acids and Its Role in Regulation of Cell Survival and Apoptosis. Int. J. Mol. Sci..

[59] Dash S., Dash C., Pandhare J. (2021). Activation of proline metabolism maintains ATP levels during cocaine-induced polyADP-ribosylation. Amino Acids.

[60] Calipari E.S., Beveridge T.J., Jones S.R., Porrino L.J. (2013). Withdrawal from extended-access cocaine self-administration results in dysregulated functional activity and altered locomotor activity in rats. Eur. J. Neurosci..

[61] Gozzi A., Tessari M., Dacome L., Agosta F., Lepore S., Lanzoni A., Cristofori P., Pich E.M., Corsi M., Bifone A. (2011). Neuroimaging Evidence of Altered Fronto-Cortical and Striatal Function after Prolonged Cocaine Self-Administration in the Rat. Neuropsychopharmacol. Off. Publ. Am. Coll. Neuropsychopharmacol..

[62] Nicolas C., Tauber C., Lepelletier F.-X., Chalon S., Belujon P., Galineau L., Solinas M. (2017). Longitudinal Changes in Brain Metabolic Activity after Withdrawal from Escalation of Cocaine Self-Administration. Neuropsychopharmacol. Off. Publ. Am. Coll. Neuropsychopharmacol..

[63] Nezhyva M., Shahen-Zoabi S., Kabirova M., Bentov-Arava E., Shalev O., Andrén P.E., Thornton C., Yaka R., Margulis K., Jansson E.T. (2024). Spatial multiomic insights into acute cocaine exposure. PNAS Nexus.

[64] Livak K.J., Schmittgen T.D. (2001). Analysis of relative gene expression data using real-time quantitative PCR and the 2(-Delta Delta C(T)) Method. Methods.

